# Therapeutic Use of Non-invasive Brain Stimulation in Dystonia

**DOI:** 10.3389/fnins.2017.00423

**Published:** 2017-07-25

**Authors:** Angelo Quartarone, Vincenzo Rizzo, Carmen Terranova, Alberto Cacciola, Demetrio Milardi, Alessandro Calamuneri, Gaetana Chillemi, Paolo Girlanda

**Affiliations:** ^1^Department of Biomedical, Dental Sciences and Morphological and Functional Images, University of Messina Messina, Italy; ^2^Centro Neurolesi Bonino Pulejo (IRCCS) Messina, Italy; ^3^Department of Clinical and Experimental Medicine, University of Messina Messina, Italy

**Keywords:** dystonia, neuroplasticity, transcranial magnetic stimulation, basal ganglia, non-invasive brain stimulation

## Abstract

Repetitive transcranial magnetic stimulation (rTMS) and transcranial direct current stimulation (tDCS) are non-invasive methods for stimulating cortical neurons that have been increasingly used in the neurology realm and in the neurosciences applied to movement disorders. In addition, these tools have the potential to be delivered as clinically therapeutic approach. Despite several studies support this hypothesis, there are several limitations related to the extreme variability of the stimulation protocols, clinical enrolment and variability of rTMS and tDCS after effects that make clinical interpretation very difficult. Aim of the present study will be to critically discuss the state of art therapeutically applications of rTMS and tDCS in dystonia.

## Introduction

Dystonia can be defined as a “movement disorder characterized by sustained or intermittent muscle contractions causing abnormal, often repetitive, movements, postures, or both” (Albanese et al., 2013).

Dystonia encompasses a heterogeneous group of syndromes that can be classified per the anatomical distribution in: focal, segmental, multifocal, hemidystonia, and generalized dystonia.

In addition, according to the etiology, dystonia can be categorized in inherited (i.e., autosomal dominant, recessive, X-linked, or mitochondrial), acquired (i.e., vascular, iatrogenic, neoplastic, traumatic, or psychogenic) and idiopathic (sporadic or familiar) (Albanese et al., 2013).

The pathophysiology of dystonia remains highly speculative although clinical heterogeneity suggests that it may be a multifactorial disease.

The paucity of symptomatic animal models is one of the reason why dystonia pathophysiology remains largely obscure (Raike et al., 2005).

Recent developed symptomatic animal models have also established the critical role of the cerebellum in dystonia, suggesting that basal ganglia and cerebellum are nodes in an integrated network that is dysfunctional in dystonia (Wilson and Hess, 2013; Richter and Richter, 2014; Pappas et al., 2015). Dystonia treatment can only partially alleviate symptoms and mainly relies on the injection of botulinum toxin in the hyperactive muscles, while the use of levodopa, anticholinergic and antiepileptic drugs has been proven to be largely ineffective (Albanese et al., 2015).

Deep brain stimulation (DBS) of the internal portion of globus pallidus (GPi) is the gold-standard of functional neurosurgical interventions for dystonia in the most severe patients and there are several evidences providing its efficacy and safety (Moro et al., 2017). On the other hand, it remains an invasive procedure so that alternative treatments are needed (Albanese et al., 2015).

In the last few years, gamma-knife and focused ultrasound lesions, which do not require surgical incision of the skull, have challenged the routine application of both the classic radiofrequency lesions and DBS. However, the application of dystonia is very limited (Higuchi et al., 2016).

Finally, transcranial magnetic stimulation (TMS) has been used in the last 20 years to explore non-invasively cortical excitability, shedding also important new insights into the pathophysiology of dystonia (Quartarone and Hallett, 2013).

In addition, TMS is a valuable technique that can be potentially used for diagnostic and therapeutic purposes in dystonia. However, the inter-subject variability in the TMS after-effects and the different pathophysiological mechanisms in the different form of dystonia, have limited diagnostic and therapeutic applications. Nevertheless, TMS can be used to differentiate between organic and psychogenic dystonia (Quartarone et al., 2009).

TMS has been proposed as noninvasive treatment in focal hand dystonia, where pharmacological options or injections of botulinum toxin are often ineffective. Finally, TMS can be considered as an adjuvant treatment in patients with cervical dystonia in conjunction with botulinum remaining the gold standard of treatment.

Hence in the present narrative review, we will describe how TMS can be used as therapeutic tool in dystonia in comparison with other noninvasive brain techniques such as transcranial direct current stimulation (tDCS).

## Non-invasive brain stimulation techniques

TMS and tDCS can stimulate the cerebral cortex painlessly through the intact skull and can produce long lasting changes in cortical excitability.

TMS was originally conceived as a non-invasive method to test the efficiency of motor pathways from the cortex to spinal cord (Rothwell, 1997).

Several experimental evidences suggest that TMS activates axons of the excitatory and inhibitory interneurons that synapse into pyramidal output neurons. In this way, the responsiveness to TMS may represent an indirect measure of the excitability of intrinsic cortical circuits. TMS can also produce long lasting changes in cortical excitability when the pulses are delivered in a repetitive fashion (Siebner and Rothwell, 2003).

Several protocols of non-invasive brain stimulation (NIBS) have been used in the last 20 years, the most common of whom are: repetitive TMS (rTMS), theta-burst stimulation (TBS) and tDCS. In all these cases, electromyography (EMG) amplitude of the motor evoked potentials (MEP) in response to single TMS stimulus is used as read out of the induced cortical plasticity.

The after effects of rTMS depend on the frequency of stimulation employed: if the pulses are given at frequency of 5 Hz or higher they facilitate excitability, whereas at frequency of 1 Hz, or lower, they depress excitability for at least 30–60 min (Quartarone et al., 2006). Thetaburst stimulation (TBS) is a protocol translated from animal studies characterized by repetitive sequences where short bursts are applied in the frequency range of EEG theta rhythms.

There are two main protocols i) the intermittent TBS (iTBS) which has facilitatory effects and ii) the continuous TBS (cTBS) which instead produces inhibitory effects. Their effect can be long lasting, up to 1 h, after the end of the conditioning protocol (Huang et al., 2005).

tDCS takes advantage of a weak polarizing direct current (1–2 mA) applied via small electrodes on the intact scalp. Several experimental evidences suggest that this small current is sufficient to polarize neurons changing their firing frequency. Anodal stimulation tends to increase cortical excitability while cathodal tends to decrease it (Nitsche and Paulus, 2001).

NIBS has been used to explore therapeutic opportunities in a bewildering variety of neurological conditions. It is now clear that, in order to get more tangible clinical effects, repeated rTMS sessions are needed (Khedr et al., 2005).

The mechanisms of action of TMS responsible for the long-lasting effects on cortical excitability are still sketchy. Changes in the effectiveness of synapses between cortical neurons such as long term depression (LTD) and long term potentiation (LTP) have been postulated based on pharmacological studies in humans. Indeed, the after effects of rTMS are abolished by a single dose of the NMDA antagonist dextromethorphan (Stefan et al., 2002). Similarly, another NMDA-antagonist, the memantine can block the after effects of some rTMS protocols (Huang et al., 2007). In addition the LTD-like depression produced by PAS10 is abolished by nimodipine, an L-type voltage-gated-Ca^2+^-channel blocker (Weise et al., 2017). Finally, several evidences suggest that TMS modulation of BDNF-TrkB pathway could play a permissive role in determining the NMDA dependent after-effects on synaptic plasticity (Wang et al., 2011).

## Pathophysiology of dystonia and therapeutic NIBS

Since dystonia etiology is very heterogeneous, dystonia pathophysiology can be a very complex puzzle (Marsden et al., 1985).

Despite the basal ganglia have been traditionally involved in dystonia, several evidences in animal models and in humans studies suggest that dystonia can be considered a network disorder (Quartarone and Hallett, 2013).

However, although it is tempting to locate the neuronal damage to a single node of the cortico-sub-cortical loop, there are now compelling evidences suggesting that, in a network perspective, it is also important to consider how remote healthy nodes of the brain may react and rearrange themselves in response to the primary damage. Such plastic reorganization may be either adaptive, compensatory, or maladaptive thus worsening the deficit (Quartarone and Hallett, 2013).

In keeping with this hypothesis, increased glucose metabolism over the striatum and anatomically related cortical motor regions such as supplementary motor area (SMA), lateral premotor cortex (PMC), anterior cingulate cortex (ACC) and dorsolateral prefrontal cortex (DLPCF) have been reported (Lerner et al., 2004; Asanuma et al., 2005).

However, several evidences suggest also an involvement of the cerebellar cortex and its direct connections with the basal ganglia and the motor cortex (Neumann et al., 2015; Cacciola et al., 2016, 2017; Milardi et al., 2016). This hypothesis is also supported by some neurophysiological data showing an abnormal cerebellar modulation over motor cortex in dystonic patients (Brighina et al., 2009).

Since dystonic patients have not overt cerebellar signs such as incoordination, loss of balance or falling, it has been postulated a compensative role of cerebellum thus pointing it out as a good candidate for therapeutic neuromodulation.

## Therapeutic approaches in dystonia: state of art

In keeping with the pathophysiological considerations discussed above, NIBS has been applied over primary motor cortex (M1), PMC, ACC and the cerebellar cortex which are important relays of the cortico-striatal and cerebello-thalamic loops.

Since TMS affects the superficial layers of the cerebral cortex it is unlikely that it may stimulate directly basal ganglia structures. On the other hand, it has been demonstrated that rTMS over the human PFC may exert remote effects on the ipsilateral caudate nucleus via a cortico-striatal release of dopamine (Strafella et al., 2001). In addition rTMS over M1 induces a reduction in raclopride binding in the left putamen if compared with rTMS of the left occipital cortex (Strafella et al., 2003).

Therefore, it can be hypothesized that some of the potential therapeutic action in movement disorders are mediated by remote sub-cortical effects.

One possible strategy in dystonia is an increase of inhibitory mechanisms. In keeping with this hypothesis, it has been reported that 30 min of inhibitory low frequency stimulation over M1 may reduce writing pressure for at least 3 h in patients with focal hand dystonia (FHD) (Siebner et al., 1999).

Similarly, 1 Hz rTMS over PMC improved handwriting velocity and hand discomfort during writing (Tyvaert et al., 2006). In addition, the effect of rTMS was compared in three different motor areas including PMC in patients with FHD. This study revealed that rTMS (20 min 0.2 Hz rTMS) over PMC is more effective than M1 and SMA repetitive stimulation (Murase et al., 2005). The clinical effects were paralleled by increased cortical inhibition as indexed by a prolonged cortical silent period (Murase et al., 2005). In the same study the authors did not report any therapeutic effect of rTMS over M1 (Murase et al., 2005); the discrepancy with the study of Siebner could be due to the different parameters of stimulation (Siebner et al., 1999).

A similar beneficial effect was obtained in a subsequent study employing cTBS over the left PMC which however did not restore deficient inhibitory mechanisms (Veugen et al., 2013).

The beneficial effects of PMC stimulation are in keeping with an open trial of epidural PMC stimulation after at least 1 month of stimulation (Lalli et al., 2012).

Another study has used rTMS over PMC (Lefaucheur et al., 2004), however the lack of a placebo arm makes the interpretation of data very difficult. In this study the authors applied inhibitory rTMS over PMC for 5 consecutive days in patients with generalized secondary dystonia showing a significant clinical effect as indexed by the reduction of the Burke-Fahn-Marsden scale (Lefaucheur et al., 2004).

It is interesting to note that the parameters of cortical excitability, tested with TMS, can be used as prognostic markers of response to rTMS. For instance, it has been reported that only patients with a modulation of cortical inhibition do respond to rTMS treatment (Kimberley et al., 2015).

Altogether, these data suggest a potential therapeutic role of rTMS over PMC. The efficacy of PMC neuromodulation is not surprising considering that PMC is implicated in sensory-motor integration and motor learning.

Another potential target of stimulation is the somatosensory cortex (SCC). It has been indeed shown that 5 Hz rTMS may enhance tactile discrimination in healthy subjects (Ragert et al., 2003). In addition, it has been widely reported that patients with FHD have significant alterations of sensory-motor integration (Quartarone et al., 2003) as well as distortion of the fingers representation map in SCC (Butterworth et al., 2003).

High frequency stimulation over SCC is not beneficial in FHD, with no effect on tactile discrimination in comparison to controls (Schneider et al., 2010). In addition, it is interesting to note that the improvement of tactile discrimination in healthy controls after rTMS, was associated with an increased connectivity in the stimulated SCC, bilateral PMC and basal ganglia, which was not the case in FHD. Therefore, it can be postulated that a cortical-subcortical disconnection may be the basis of the ineffectiveness of rTMS (Schneider et al., 2010).

In another placebo controlled study, low frequency 1 Hz stimulation was delivered over SCC 30 min per day for 4 consecutive weeks in 15 patients affected by writer's cramp (Havrankova et al., 2010). The procedure was successful only in 4 out of 15 patients and was strictly related to the precise coil localization of a narrow strip over the post central sulcus (Havrankova et al., 2010).

ACC has been used as another potential target since this area has an increased activation with PET studies in patients with blepharospasm (Ceballos-Baumann and Brooks, 1998; Kerrison et al., 2003). Low frequency stimulation (0.2 Hz), delivered in a randomized controlled study, can significantly reduce eye blink rate, the number of sustained blinks and the time to eye closure (Kranz et al., 2009). This clinical effects was associated with a normalization of the blink recovery cycle (Kranz et al., 2010).

A new appealing target for NIBS is cerebellum since several evidences suggest that the cerebellum may play a compensatory role in dystonia (Jinnah and Hess, 2006; Quartarone and Hallett, 2013).

In a randomized controlled study, iTBS was delivered bilaterally over the cerebellum for 5 consecutive days for 2 weeks in 20 right handed patients affected by cervical dystonia.

This protocol induced a transient improvement of dystonia and was paralleled by a restoration of the topographic specificity of PAS with a disappearance of the facilitation on Fist Dorsal Interosseus (FDI) (Koch et al., 2014).

On the other hand, tDCS has brought conflicting results, in one study tDCS over the cerebellum was successful in FHD (Bradnam et al., 2015), while in other study it did not work (Sadnicka et al., 2014).

Similarly cathodal tDCS tested over M1 in a randomized double blind sham-controlled study was not successful in a population of writer's cramp patients and musicians cramp (Benninger et al., 2011; Buttkus et al., 2011).

Finally, since there is an enhanced sensory-motor integration, another feasible strategy is to provide independent inputs from dystonic muscles via an asynchronous afferent stimulation avoiding any temporal coupling of the evoked afferent inputs (Schabrun et al., 2009).

The idea is that a period of asynchronous afferent stimulation or non-associative stimulation (NAS) may reverse maladaptive cortical changes and alleviate symptoms.

By using a NAS protocol consisting of asynchronous electrical stimuli (never delivered together with a random inter-stimulus interval ranging from 0.15 to 2.85 as well as with stimulus intensity set to evoke a tiny muscle contraction) applied to the motor points of FDI and abductor pollicis brevis (APB) for 1 h, it has been demonstrated in FHD patients that NAS transiently normalizes the distorted motor map and can significantly reduce movement variability during cycling drawing (Schabrun et al., 2009).

## Limitations and future perspectives

There are several factors that that strongly limit the interpretation of results after rTMS studies.

Perhaps the most important limitation is the lack of an optimal placebo condition, the so called sham condition. In theory, tilting the coil should dramatically reduce the biological effects of TMS, however several modeling studies in animals are now suggesting that tilting the coil over the skull does not exclude the possibility of a tiny cortical activation (Lisanby et al., 2001).

On the other hand, by using shield equipped coils and a tilt of 90 degrees it is possible to minimize the effective magnetic field (Duecker and Sack, 2013).

Another not risible issue is that active rTMS, besides its cortical effects, is associated with a characteristic click sound and a stimulation of trigeminal afferents. Therefore, to mimic click sound and trigeminal stimulation new dedicated sham shielded coils have been designed with a delivered magnetic field of only 10% compared to active coils.

Another limitation is that most of the studies have addressed primary dystonia while the therapeutic effect of NIBS in secondary dystonia is still unknown.

Despite therapeutic cerebellar stimulation is promising, the gold standard in dystonia is targeting the motor areas strictly connected with basal ganglia (Bharath et al., 2015).

The major limitation of all these studies are the small sample size, the presence of different phenotypes in the same cohort of patients, as well as the fact that several different parameters of stimulation have been adopted across studies.

Despite this extreme variability, it looks like that to be successful NIBS needs to be delivered in a multisession design. Some single session studies have shown positive results that however were not persistent (Murase et al., 2005; Furuya et al., 2014).

There are other possible confounding factors such as preliminary exercise, time of the day and concomitant medications (Ridding and Ziemann, 2010).

Finally, in most studies, stimulation was not performed under neuronavigation to maintain an adequate coil position during stimulation sessions.

Nevertheless, these preliminary results reinforce the idea that NIBS can represent a promising alternative therapeutic opportunity in dystonia.

Several recommendations could be considered in future therapeutic trials: first, it will be important in future studies to determine the best stimulation target, second to use multisession designs with neuronavigation and finally to increase the sample size with multicenter approaches. Another requirement is to design more efficient stimulation protocols to prolong the therapeutic effects. Last but not list, rTMS could be used, soon, to pre-select possible candidates to invasive surgical stimulation approaches.

## Author contributions

AQ: work conception and design, drafting the work, work revision, final approval, and global agreement. VR: data interpretation, work revision, final approval, and global agreement. CT, AlbC, DM, AleC: work conception and design, work revision, final approval, GC: data interpretation, drafting the work. PG: work conception and design, guarantor of integrity of entire study, manuscript revision for important intellectual content, final approval.

### Conflict of interest statement

The authors declare that the research was conducted in the absence of any commercial or financial relationships that could be construed as a potential conflict of interest.
